# Supplementary material for: Global predictions for the risk of establishment of Pierce’s disease of grapevines
Source: Commun Biol. 2022 Dec 20;5:1389. doi: 10.1038/s42003-022-04358-w (PMC9768138; doi:10.1038/s42003-022-04358-w)
Supplement: Supplementary file 3 — Description of Additional Supplementary Files [file 42003_2022_4358_MOESM3_ESM.pdf]

Description of Additional Supplementary Files

Supplementary Data 1- Results of the inoculation assays on European grapevine varieties with two isolates of *Xylella fastidiosa* subsp. *fastidiosa* (ST1) carried out in Mallorca between 2018 and 2020.

Supplementary Data 2- Distribution of Pierce's disease in the United States. Data was obtained from different publications in which at least information of the county was provided.

Supplementary Data 3- Pierce's disease risk of establishment in winegrowing regions of China and the Southern Hemisphere.

Supplementary Data 4- Pierce's disease risk areas in European vineyards given by the intersection of Corine-Land-Cover and the projected model in the ERA5-land data under a Ro=5 scenario and a spatial heterogeneous vector distribution for 2019 and projection for 2050.
